# Slow clearance of histidine-rich protein-2 in Gabonese with uncomplicated malaria

**DOI:** 10.1128/spectrum.00994-24

**Published:** 2024-08-28

**Authors:** Carlos Lamsfus Calle, Frieder Schaumburg, Thorsten Rieck, Anne Marie Nkoma Mouima, Pablo Martinez de Salazar, Saskia Breil, Johannes Behringer, Peter G. Kremsner, Benjamin Mordmüller, Rolf Fendel

**Affiliations:** 1 Institute of Tropical Medicine, University of Tübingen, Tübingen, Germany; 2 German Center for Infection Research (DZIF), Partner Site Tübingen, Tübingen, Germany; 3 Centre de Recherches Médicales de Lambaréné, Lambaréné, Gabon; 4 Institute of Medical Microbiology, University Hospital Münster, Münster, Germany; 5 Swiss Tropical and public Health Institute, Allschwil, Switzerland; 6 Department of Medical Microbiology, Radboud University Medical Center, Nijmegen, the Netherlands; Hubei University of Medicine, Shiyan, Hubei Province, China

**Keywords:** histidine-rich protein-2, malaria, *Plasmodium falciparum*, rapid diagnostic test, half-life, diagnosis

## Abstract

Malaria rapid diagnostic tests (RDTs), which detect *Plasmodium falciparum* (Pf)-specific histidine-rich protein-2 (HRP2), have increasing importance for the diagnosis and control of malaria, especially also in regions where routine diagnosis by microscopy is not available. HRP2-based RDTs have a similar sensitivity to expert microscopy, but their reported low specificity can lead to high false positivity rates, particularly in high-endemic areas. Despite the widespread use of RDTs, models investigating the dynamics of HRP2 clearance following Pf treatment focus rather on short-term clearance of the protein. The goal of this observational cohort study was to determine the long-term kinetic of HRP2-levels in peripheral blood after treatment of uncomplicated malaria cases with Pf mono-infection using a 3-day course of artesunate/amodiaquine. HRP2 levels were quantified at enrollment and on days 1, 2, 3, 5, 7, 12, 17, 22, and 28 post-treatment initiation. The findings reveal an unexpectedly prolonged clearance of HRP2 after parasite clearance from capillary blood. Terminal HRP2 half-life was estimated to be 9 days after parasite clearance using a pharmacokinetic two-compartmental elimination model. These results provide evidence that HRP2 clearance has generally been underestimated, as the antigen remains detectable in capillary blood for up to 28 days following successful treatment, influencing RDT-based assessment following a malaria treatment for weeks. A better understanding of the HRP2 clearance dynamics is critical for guiding the diagnosis of malaria when relying on RDTs.

**IMPORTANCE:**

Detecting *Plasmodium falciparum*, the parasite responsible for the severest form of malaria, typically involves microscopy, polymerase chain reaction (PCR), or rapid diagnostic tests (RDTs) targeting the histidine-rich protein 2 or 3 (HRP2/3). While microscopy and PCR quickly turn negative after the infection is cleared, HRP2 remains detectable for a prolonged period. The exact duration of HRP2 persistence had not been well defined. Our study in Gabon tracked HRP2 levels over 4 weeks, resulting in a new model for antigen clearance. We discovered that a two-compartment model accurately predicts HRP2 levels, revealing an initial rapid reduction followed by a much slower elimination phase that can take several weeks. These findings are crucial for interpreting RDT results, as lingering HRP2 can lead to false positives, impacting malaria diagnosis and treatment decisions.

## INTRODUCTION

*Plasmodium falciparum* (Pf) is still among the 10 most common leading causes of death in low-income countries, predominantly found in the Sub-Saharan region of Africa (1, 2). To this day, the main strategies in malaria control programs include insecticide-treated mosquito nets, indoor residual spraying, chemoprevention in pregnant women and children, increased use of diagnostic tests, prompt and proper treatment, and reduction in the ratio of treatments to tests (2). These interventions have resulted in a decline in malaria cases and deaths since the beginning of the century, but a plateau has been reached since about 2015, with a slight increase in disease burden since 2020 (2). Time to diagnosis is crucial for managing malaria and, therefore, an accurate diagnostic tool becomes essential. Giemsa-stained thick and thin blood smears have been the gold standard for parasite detection by microscopy for decades (3). However, in daily practice in hospitals in rural settings, this technique is not always performed well since it requires well-trained microscopists, continuous validation, and quality assurance. Otherwise, the sensitivity and specificity of this technique vary markedly between laboratories (3–5). As a result, delays may occur in initiating treatment, being especially crucial in critical cases, particularly in remote areas with limited resources (6). The development of malaria rapid diagnostic tests (RDTs) aimed to fill this gap, even though the WHO still recommends microscopic confirmation before initiating treatment (except for small children and pregnant women) (7, 8). Nevertheless, the widespread use of rapid diagnostic tests has contributed greatly in some countries to the paradigm shift of starting antimalarial treatment after obtaining a parasitological diagnosis by microscopy or rapid diagnostic tests (9).

The most common RDTs are based on the immobilization of parasite antigens by monoclonal antibodies fixed to an absorbent surface after lateral flow of a blood sample from individuals suspected of having malaria. RDTs are designed to detect one to three different antigens which are either Pf-specific (histidine-rich protein-2, HRP2) or pan-specific (pan malaria lactate dehydrogenase, pLDH, or pan malaria aldolase) (10). RDTs demand less expertise and are a time-saving alternative to microscopy.

Although RDTs in endemic areas may achieve a sensitivity similar to that of thick blood smear microscopy under optimal conditions [≥95% at ≥100 parasites per µL under routine conditions (11)], the sensitivity and specificity recommended by WHO are not always met under field conditions (5, 6, 12–15). For instance, the sensitivity of pLDH-based RDTs is often insufficient, while specificity is usually very high (>99.5%) (16, 17). Aldolase sensitivity and specificity are comparable to that of pLDH (9). The most sensitive and, therefore, most widely employed RDTs use *P. falciparum* HRP2 as the target antigen (5, 18). In addition, also new ultrasensitive HRP2-based RDTs have been developed, with even higher sensitivity (19, 20). The main drawback of HRP2-RDTs is their specificity (13). Based on these distinct detection capabilities, HRP2 and aldolase antigens were combined within the same RDT cassette to compensate for the specificity of one antigen with the sensitivity of the other (21).

One reason for the low specificity of HRP2-RDTs may be the persistence of the antigen in the circulation following treatment of *P. falciparum* malaria, as was already described in 2001 (22). This might explain the association between the specificity of the test and malaria endemicity (23, 24), which presumably contributes to the false-positive results seen in healthy individuals who have been successfully treated (12). Long-term detection of HRP2 has been proposed to be due to the persistence of sexual (25, 26) and asexual phases in the deep capillary beds (18, 27), but so far evidence is scarce. A more precise understanding of the half-life of the HRP2 parasite protein is needed due to the difficulty in determining whether antigen accumulation results from recurrent and recent infections or a constant low parasitemia only detectable by polymerase chain reaction (PCR) but not by microscopy. Reported half-lives to date were calculated from measurements taken in the first few days post-parasite treatment (24, 28, 29). However, several studies have reported positive HRP2-RDTs for more than 28 days after treatment (26, 30, 31).

In the study presented here, 27 participants with uncomplicated falciparum malaria were treated and followed up to determine the HRP2 decay over a period of 28 days (32). The false positivity rate of aldolase- and HRP2-RDTs relative to parasitemia detected by microscopy and qPCR was calculated. The half-life of HRP2 was assessed using repetitive quantitative measurements of HRP2 by ELISA, to generate a model for the decay of the parasitic protein.

## MATERIALS AND METHODS

### Study design

Subjects aged between 6 months and 25 years with fever in Lambaréné region (Gabon) between January and March 2009 were screened for eligibility to participate in the study. Participants were enrolled when they met the following inclusion criteria: (i) diagnosed uncomplicated Pf malaria (excluded mixed infection) and (ii) acute fever or history of fever in the last 48 hours.

Participants showing symptoms or signs of severe malaria [according to the WHO definition (7, 8)] or who had participated in any vaccine trial or had received any antimalarial in the last 28 days or who were suspected of having any other disease, were excluded and referred to the appropriate specialist if necessary. Any comorbidity was treated according to the guidelines.

Eligible participants received a 3-day oral treatment of artesunate/amodiaquine (Coarsucam, Sanofi-Aventis), namely at recruitment (day 0), day 1, and day 2 of follow-up. Since 2003, artesunate/amodiaquine has been a first-line treatment for children and adults with uncomplicated malaria caused by Pf in Gabon (33–35). The first dose of artesunate/amodiaquine was calculated according to age or weight and was administered after the first blood collection (day 0). Follow-up visits were conducted at home on days 3, 5, 7, 12, 17, 22, and 28 post-treatment initiation.

During the first 3 days of treatment and subsequent visits, 150 µL whole blood samples were collected in EDTA capillary tubes (KABE Labortechnik, Nümbrecht-Elsenroth, Germany). Capillary blood was stored at −20°C immediately after sampling and used to determine HRP2 levels and to determine parasitemia by qPCR.

### Parasitemia

A Giemsa-stained thick blood smear (TBS) from the fingertip of each participant in each visit was read using the Lambaréné method (36) by two independent microscopists.

Briefly, a total of 20 microscopy fields were counted if parasitemia was ≥50 Pf parasites per field, 30 fields if parasitemia was 5–50 Pf parasites per field, and 100 fields if parasitemia was ≤5 Pf parasites per field. The slide was considered negative if no parasite was found in 100 fields. A third microscopist read the slide in case the results were divergent on positivity/negativity and when there was more than 25% discordance (lower value/upper value <0.75) in the results of asexual and sexual counts. If so, the mean parasitemia of the two closest parasite concentrations was used.

### HRP2 enzyme-linked immunosorbent assay

For quantitative detection of HRP2, a sandwich ELISA was conducted as previously described with minor modifications (37). A 96-well high-binding flat-bottom microtiter plate (Microlon 600, highbinding, F-Boden, Greiner Bio-One GmbH, Frickenhausen, Germany) was coated with 1 µg/mL anti-PfHRP2 IgM antibody (Immunology Consultants Laboratories, Inc., Newberg, USA) in PBS by overnight incubation at 4°C followed by a blocking step using 2% BSA in PBS. All subsequent washing steps were done using PBS supplemented with 0.05% Tween-20. Hemolyzed whole blood samples were incubated at room temperature for 1 hour in three different dilutions: 1:50, 1:100, and 1:200. The standard curve was prepared by twofold serial dilutions from a complete culture of *P. falciparum* 2% parasitemia/5% hematocrit (1:4 to 1:4,096). All samples and standard curve dilutions were plated in duplicates. After 1-hour incubation at room temperature with 0.2 µg/mL anti-PfHRP2 IgG detection antibody (Immunology Consultants Laboratories, Inc., Newberg, USA), plates were developed with TMB chromogen (Zymed Lab., Inc., San Francisco, CA, USA) and stopped with 1 M sulfuric acid. Absorption was measured at 450 nm with an ELISA reader (Asys Expert 96, anthos Mikrosysteme GmbH, Krefeld, Germany). Results were expressed as arbitrary units (AU) relative to the amount of the undiluted parasite culture used as standard.

### *Plasmodium spp.* antigen detection by rapid diagnostic test

EDTA capillary blood was used for RDTs according to the manufacturer’s instructions (Paracheck-Pf, Orchid Biomedical Systems, Verna, India, and BinaxNowMalaria, Binax Inc., Inverness Medical, Scarborough, Maine, USA). RDTs were performed at the same time as the TBS. When a participant obtained negative RDT results at two consecutive time points from day 0, the performance of any subsequent RDT was suspended. The test was considered invalid if the control band was not stained. The RDT was repeated twice in case the test result was invalid. BinaxNow consisted of three detection bands: (i) Control, (ii) PfHRP2, and (iii) Pan-*Plasmodium spp.* Aldolase while Paracheck-Pf presented only two bands (i) PfHRP2 and (ii) Control. Paracheck-Pf can only determine whether participants are infected with Pf, whereas BinaxNow detects species beyond Pf, as it has the dual ability to detect the pan-*Plasmodium* aldolase antigen (T2 band) together with HRP2 (T1 band).

### DNA extraction and 18s ribosomal gene amplification by RT-qPCR

DNA extraction was performed according to the manufacturer’s instructions with a QIAamp DNA mini kit (Qiagen, Hilden, Germany). PhHV-1 was co-extracted with each sample acting as a DNA extraction control and PCR control.

Primers and probes for amplification of the 18S rRNA gene locus and the PhHV-1 control fragments are shown in Table S1. Plasmid isolations containing the small subunit (SSU) rRNA gene sequence from either *P. falciparum*, *P. malariae*, *P. ovale*, or *P. vivax* were used as *Plasmodium spp*. specific controls.

Optimization of primer concentration to limit interactions was implemented. The lowest primer concentration was selected at which the Ct obtained did not show a relevant increase compared to the Ct values of higher primer concentrations.

For all amplification reactions, the Rotor-Gene 6000 PCR thermocycler (Corbett, Australia) was used with a 25 µL volume containing 1× HotstarTaq master mix PCR buffer, 3.5 mM MgCl_2_, 2.5 µg BSA, varying amounts of primers (see below), 100 nM Taqman probe, and 5 µL DNA sample.

DNA was denatured for 15 min at 95°C followed by 50 cycles of 15 s at 95°C and 60 s at 60°C with fluorescence data acquisition at the 60°C step. The gain of the photomultiplier was manually adjusted to 8, 10, 10, and 8 for the green (FAM fluorophore), yellow (VIC), orange (ROX), and red (Cy5) channels, respectively.

Thresholds were set within the exponential phase of the amplification curves of the PhHV-1 as well as the plasmodial (SSU) rRNA genes and adjusted for internal positive controls. A triplicate dilution series of capillary blood from a patient with microscopically confirmed Pf malaria served for the standard curve generation.

If any of the species-specific positive controls were negative or there was an amplification signal from the negative controls in any of the parasite channels, the PCR result was excluded from data analysis, and sample preparation and PCR were repeated. Samples in which the Ct value of the PhHV-1 internal control differed by more than 2 Ct values from the median in each series were omitted from the analysis and reprocessed.

### Genotyping

Genotyping was based on the amplification of MSP-1, MSP-2, and GLURP polymorphic genes in those patients who presented recurrent parasitemia after antimalarial treatment, as previously described (38, 39).

### Two-compartment model

The two-compartment model is composed of one central compartment (circulating plasma levels) and a second compartment (which is usually organs or peripheral tissues) in which the compound can accumulate. The system seeks an equilibrium between the two compartments regulated by constants k_12_, which controls the distribution of the drug to the second compartment, and a constant k_21_ when distribution occurs between the second and the central compartment. When there is an equilibrium between the second and the central compartment, elimination (k_el_) follows a first-order process. This constitutes the terminal plasma half-life, defined as the time required to reduce the plasma concentration by half after reaching a pseudo-equilibrium. Before this happens, the half-life can be misleading. This elimination model is usually applied to intravenous drugs (Fig. S1a).

The two-compartment elimination model becomes clear when a straight line (Fig. S1b and c) is plotted for the last time points on a semilogarithmic plot of the logarithm of HRP2 values (y-axis) to days (x-axis). The slope of the generated line (*β*) is needed to calculate what is called the terminal half-life of the protein, by using the formula:


(1) $$t^{\frac{1}{2}}=\frac{Ln\left(2\right)}{\beta }$$

The subtraction of the actual HRP2 values during the first time points and the values calculated from the freshly generated regression line (purple line, Fig. S1c and d) for the same days generate the values called “residuals.”


(2) $$Residuals=HRP2-HRP2^{late}=A\times \;e^{-\propto \;t}$$

These values represent an elimination closer to reality during the initial elimination phase before equilibrium is reached in the organism. When a linear regression is calculated from these values (Fig. S1d and e), a new slope (α) is generated which helps to calculate the initial extrapolated half-life with the formula:


(3) $$t^{\frac{1}{2}}=\frac{Ln\left(2\right)}{\alpha }$$

By using α and β in conjunction with the intercept on the y-axis of each regression line, the constants k_21_, k_12_, and k_el_ can be determined through the following equations:


(4) $$k21=\;\frac{A\;\times \beta +B\times \;\propto }{A+B}$$

(5) $$kel=\;\frac{\propto \;\times \;\beta }{k21}$$

(6) k12= ∝ + β−k21−kel

### Statistics

Shapiro-Wilk test and Kolmogorov-Smirnov test were used to test for normal or log-normal distribution of the continuous data variables. All correlations were conducted using Spearman’s rank order test. The result was considered statistically significant when the *P*-value was below 0.05 and the absolute value of the correlation coefficient was equal to or higher than 0.5.

## RESULTS

### Study development

A total of 27 participants presenting with Pf mono-infection (determined microscopically and subsequently confirmed by 18S rRNA qPCR) were enrolled in the present study. All participants experienced at least fever as a symptom of malaria in the 48 hours prior to inclusion but did not show any symptoms related to severe malaria (Fig. S2a; Table S2). All but one study participant completed the follow-up period. The guardian of one child withdrew consent on day 22 but agreed to the use of the data already collected.

**Fig 1:**
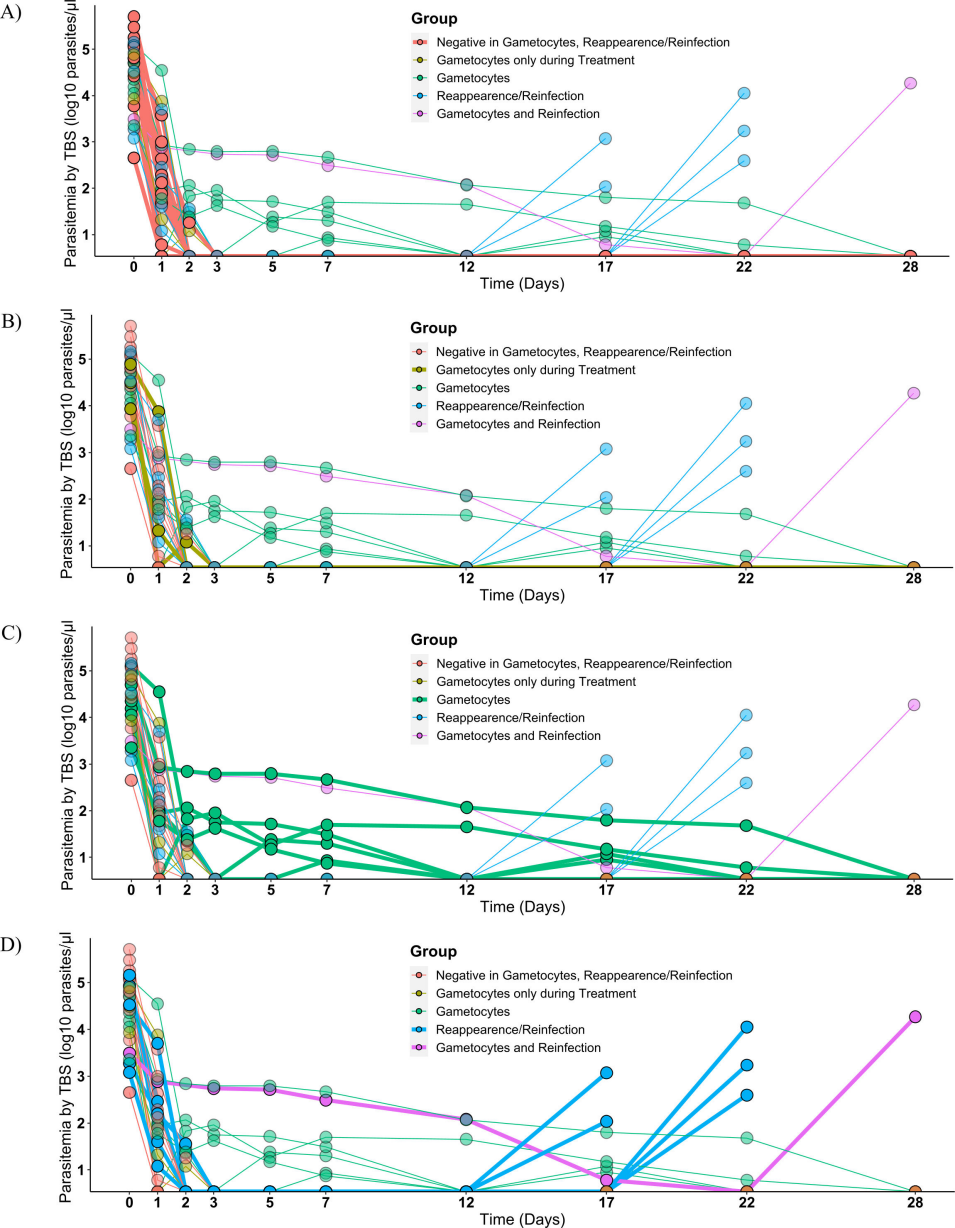
Parasite clearance from first artesunate/amodiaquine administration. Parasitemia was estimated for 28 days by microscopy after drug administration (artesunate/amodiaquine) at day 0. The kinetics of the parasitemia is illustrated, with thicker lines highlighting the different groups, as estimated by TBS for the full follow-up period for participants not having gametocytes or reappearance/reinfections (**A**), those with only gametocytes during the treatment phase (**B**), gametocytes carriers during several time points after treatment, (**C**) and the participants that suffered reinfections and reappearances (**D**). Of note, one patient experienced a reappearance on day 22, five participants experienced reinfection, and from those, one also presented circulating gametocytes. In total, 10 participants showed circulating gametocytes during the trial, of those, three during the treatment phase only.

The median parasitemia measured by TBS at inclusion was 33,435 parasites/µL (range 449–508,200 parasites/µL; Fig. 1; Fig. S3). In the 24 hours post-administration of the first dose of treatment (day 1), nine participants became TBS negative while the median parasitemia decreased to 145 parasites/µL (range 6–35,220 parasites/µL) in those who remained positive. On day 2, only 2 out of 27 (7%) participants remained positive. Both had low parasite counts (18 and 36 parasites/µL). A higher initial parasitemia at inclusion was found in participants who still presented circulating parasites on day 1 than in those who cleared parasites 24 hours post-drug administration (median parasites/µL 71,118 and 8,525, respectively; Wilcoxon test *P* < 0.01).

Following the completion of treatment on day 2, participants underwent follow-up on days 3, 5, 7, 12, 17, 22, and 28 after inclusion (Fig. S2b). Among the cohort of 27 participants, a subset of six individuals (6/27, 22%) exhibited reemergence of asexual parasites under microscopic examination after a span exceeding 12 days (see Fig. 1D). Specifically, this resurgence occurred in two patients on day 17, on day 22 in three patients, and day 28 in one patient. Five of these cases were classified as reinfections, while one was identified as a recrudescence of the initial infection, as evidenced by amplification-based genotyping (data not shown). This indicates a treatment failure rate of 1 out of 27 participants (4%).

Notably, the recrudescence event was observed on day 22.

Ten participants presented microscopically detectable gametocytes during follow-up, of which three showed detectable circulating sexual stages (9–21 parasites/µL) only during the treatment phase (Fig. 1B and C; Fig. S4a). The median gametocytemia across all patients during follow-up was 46 parasites/µL (range 6–1,890 gametocytes/µL) detected for up to 23 days (mean 12 days; 95% CI: 6–18).

During the first 3 days of treatment, the decreases in parasitemia measured by qPCR and microscopy were very similar. The applied qPCR approach is unable to discriminate between the asexual and sexual stages of the parasite. Thus, if all gametocyte carriers were excluded the parasitemia decreased from 32,461 parasites/µL on day 0 to 457 parasites/µL on day 1 and 45 parasites/µL on day 2 (Fig. S4b). The two participants with the lowest parasitemia in qPCR (157–197 parasites/µL) at inclusion had undetectable parasitemia from day 1 onwards. All participants were determined to be free of asexual parasites by qPCR beyond day 12, with the only exception being that gametocyte carriers after completion of treatment were still positive past this point as were those who showed reappearance/reinfections (Fig. S4b). The participants who experienced reinfections or recrudescence during the trial period were confirmed by qPCR (Fig. S4b).

### HRP2

Circulating HRP2 in whole blood was quantified by ELISA. The baseline HRP2 level at inclusion for all participants was 2.48 AU (95% CI: 1.38–4.44). Initial parasitemia showed a positive correlation with HRP2 levels (Spearman *P* < 0.01, correlation coefficient 0.6) at day 0. The participants who presented the highest parasitemia at inclusion had early HRP2 levels above the upper limit of detection of the ELISA method. The two participants with the lowest parasitemia were below the lower limit of detection (0.01 AU) as early as day 1, rendering later half-life calculations for them impossible. Samples from two additional participants were not available to perform HRP2 ELISA. However, both participants showed gametocytes at several points following treatment. No association was found between the presence of gametocytes either at the beginning or during the study and the initial among of HRP2 (data not shown). Gametocytes could still be detected in four participants after day 12, although the HRP2 ELISA remained below the lower detection limit. Participants experiencing reinfections or recrudescence showed an identifiable increase in HRP2 levels when TBS became positive (2.3 AU 95% CI: 0.5–4.1).

**Fig 2:**
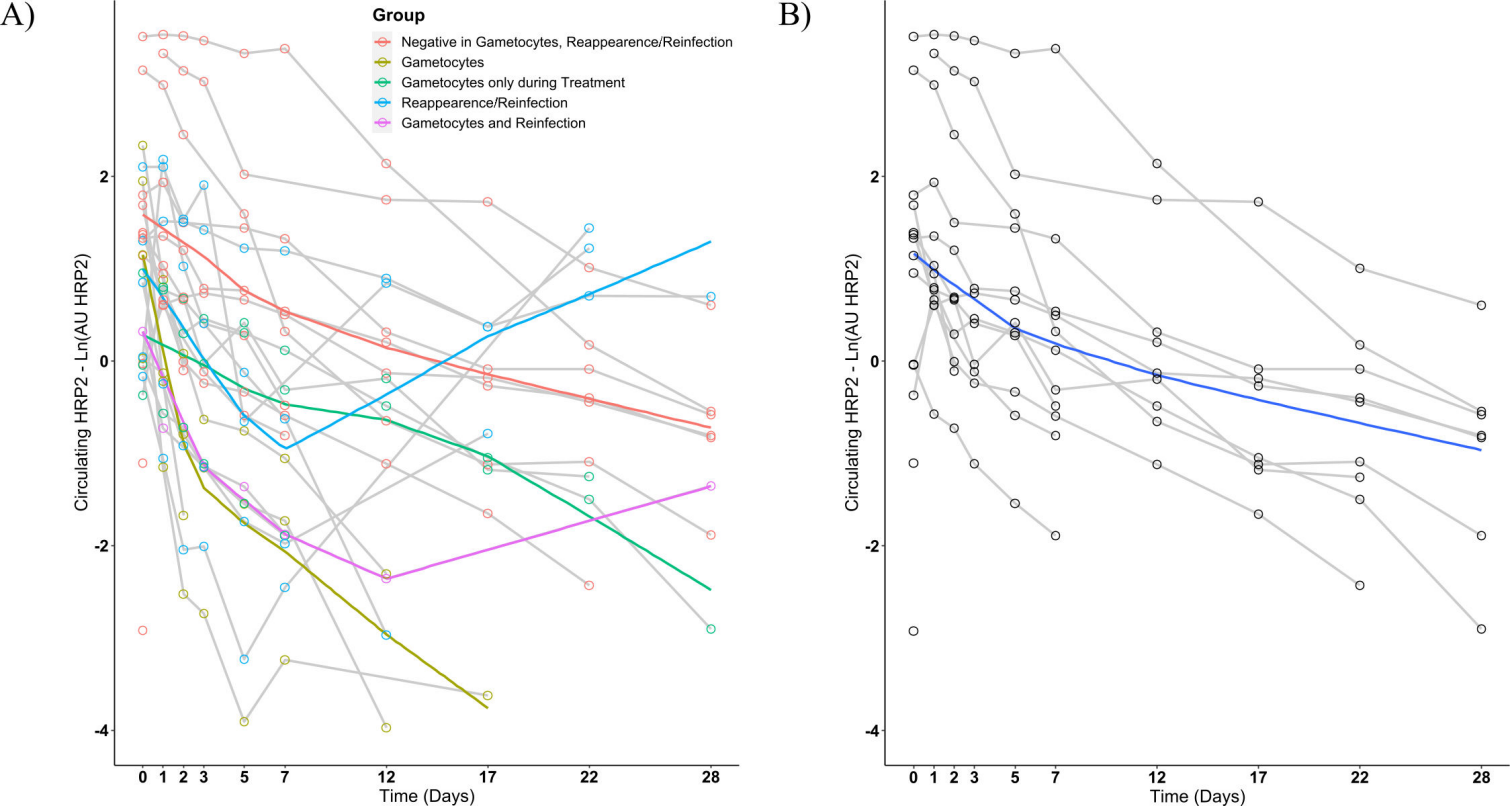
HRP2 concentration during the follow-up period. HRP2 concentration in the peripheral blood is represented as a semi-logarithmic graph for the period from d0 to d28 (*N* = 25) including all participants (**A**) or excluding those with either *Plasmodium* spp. reappearances or reinfections and those with prolonged gametocytemia after treatment (**B**). Each line represents the kinetic for the HRP2 measure of a study participant. The colored lines in graph (**A**) represent the lowess (locally weighted scatterplot smoothing) regression for the different groups reflected on the legend. The blue line in graph (**B**) represents a lowess regression of the presented data set.

Circulating HRP2 concentration decreased more slowly during the study than parasitemia regardless of parasites being determined by microscopy or qPCR (Fig. 2). This was not explained by the cases of recrudescence or reinfections nor by the presence of gametocytes. On day 28, there were still seven positives for HRP2 that did not belong to the gametocyte-positive group and did not have recrudescence or new infections. Initial parasitemia was highly correlated with the time until a negative HRP2-ELISA test was obtained (Spearman *P* < 0.01, correlation coefficient 0.6) and was associated with the levels of HRP2 for each timepoint (Spearman *P* < 0.01, correlation coefficient D1 0.9; D2 0.9; D3 0.8; D5 0.8; D7 0.6; D12 0.8; D17 0.6; D22 0.6; and D28 0.6). One day after the start of treatment, seven participants experienced an increase in HRP2 levels despite the decrease of parasitemia in the peripheral blood as shown by TBS.

### Half-life HRP2

Despite the steep decline in circulating parasites during the first 2–3 days, HRP2 levels only decrease slightly during this time (Fig. 2). However, there is a clear initial HRP2 curve that constitutes an initial clearance. Once parasites are no longer detected, HRP2 concentration should only increase in the case of reinfections or recrudescence. Therefore, to avoid interferences, participants undergoing new infections or recrudescence were excluded from the analysis together with those showing gametocytes from day 3 to day 28. The participants who had gametocytemia only during the treatment phase were not excluded from further analysis, as gametocytemia was low and no gametocytes were detected again.

For six participants with lower parasitemia at inclusion, HRP2 levels were below the limit of detection already by day 12 of the study, reducing the information on the long-term behavior of the protein. Thus, the remaining nine participants were selected for the terminal half-life calculation (Fig. S5).

**Fig 3:**
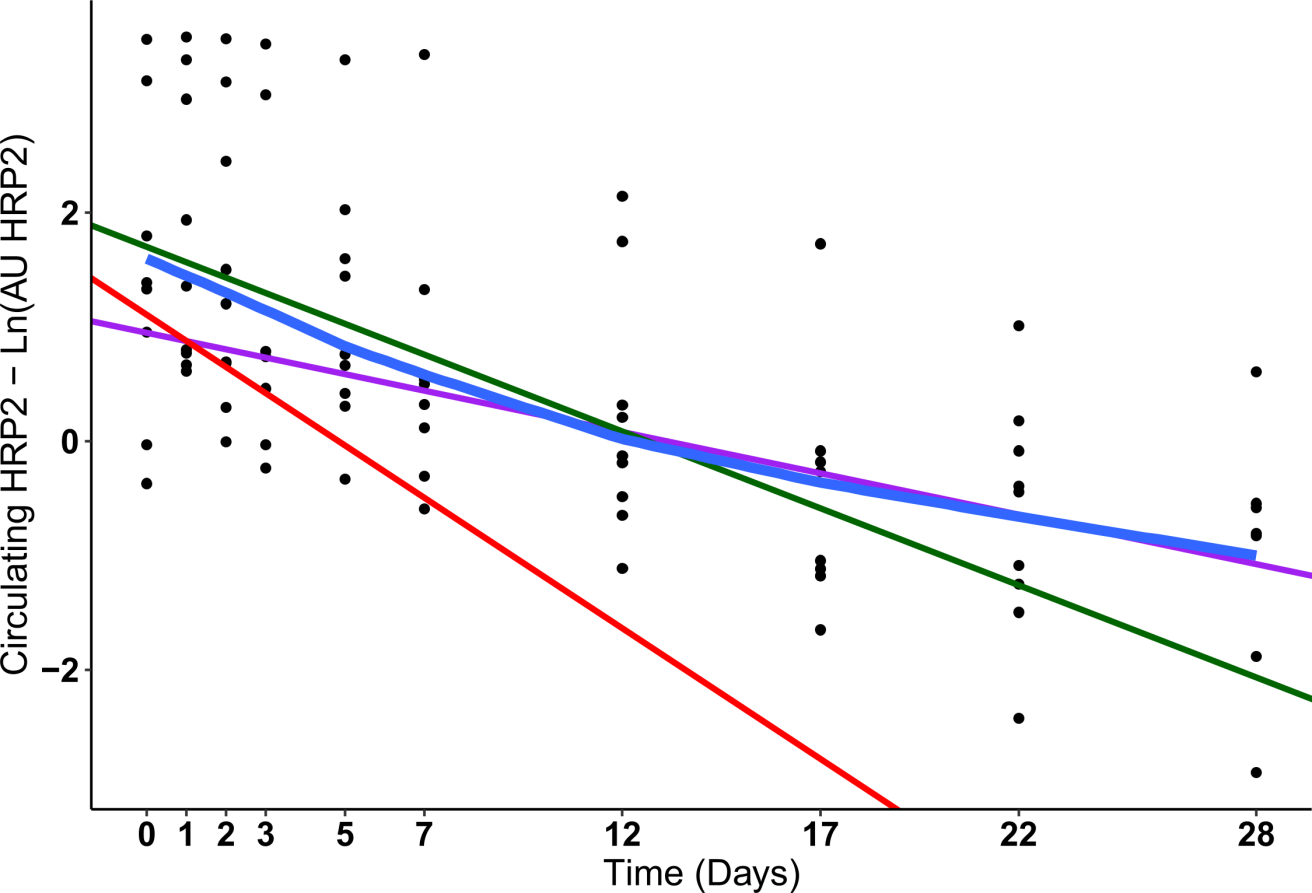
Short- and Long-term decay of HRP2. The concentration of HRP2 in the capillary blood of patients without gametocytes after treatment, no recrudescence or reinfection, and detectable HRP2 levels for more than 7 days post-treatment initiation were selected for the generation of the long- and short-term models (*N* = 9). The blue line represents a lowess regression (locally weighted scatterplot smoothing). The purple line is the linear regression from the HRP2 values at day 12, day 17, day 22, and day 28 that shows the terminal elimination. The green line is the linear regression from the HRP2 values on day 0, day 1, day 2, day 3, day 5, and day 7 showing a mix of the initial parasite elimination and the terminal elimination. The red line represents the corrected initial HRP2 elimination after subtracting the contribution of the terminal elimination.

From day 12, HRP2 elimination follows a first-order elimination, since the blood-concentration–time profile is linear if plotted on a semi-logarithmic plot (Fig. 3). A linear regression analysis, represented by the purple line, was performed using the HRP2 measurements from the last four collection time points. The slope (β) derived from this regression was employed to compute the terminal half-life (Formula 1).

The difference between the actual values of HRP2 during the treatment phase and the values of the regression line (purple line) at those time points generate the values termed “residuals” (Formula 2).

The linear regression slope for the residual values (red line) serves to calculate the extrapolated initial half-life during the first week, which corresponds to the initial elimination phase (Formula 3).

Hence, the mean terminal half-life (slope β) for the nine participants was 9 days (95% CI: 6–12 days) and the mean extrapolated initial half-life (slope α residuals) was calculated to be 2 days (95% CI: −1 to 6 days).

Using α and β in conjunction with the intercept on the y-axis of both regression lines allowed for the determination of the constants k_21_, k_12_, and k_el_ (Formulas 4, 5, and 6 and Fig. S1a). The resulting constant values are k_el_ 0.23 days^−1^ (5.46 hours^−1^), k_12_ 0.17 days^−1^ (4.12 hours^−1^), and k_21_ 0.22 days^−1^ (5.27 hours^−1^).

### HRP2-based rapid diagnostic test

Two RDTs were used for the study. Before treatment initiation, all participants showed a positive RDT for HRP2 (both RDTs), and 23 out of 27 participants were positive for Aldolase (BinaxNow T2 band). The four negative participants were among those with low parasitemia at inclusion (<6,000 parasites/µL).

Considering the complete course of the follow-up period of the study, in all measurements during the study, when aldolase (BinaxNow T2 band) was positive, HRP2 (BinaxNow T1 band and Paracheck-Pf RDT) was also positive, but never the other way around.

HRP2 ELISA results were compared with HRP2 band positivity in both RDTs. It was observed throughout the study that, with the exception of one participant, when ELISA test results crossed the lower limit of detection of the assay (at a blood dilution of 1:50 which was used for the ELISA method due to high background if used otherwise), the rapid diagnostic tests could remain positive for one or two more time points (data not shown). However, this exceptional participant, who belonged to the gametocyte group, showed a positive HRP2 ELISA on day 17, although on that day the band for both RDTs was negative and the BinaxNow T1 band had been negative since day 7.

**Fig 4:**
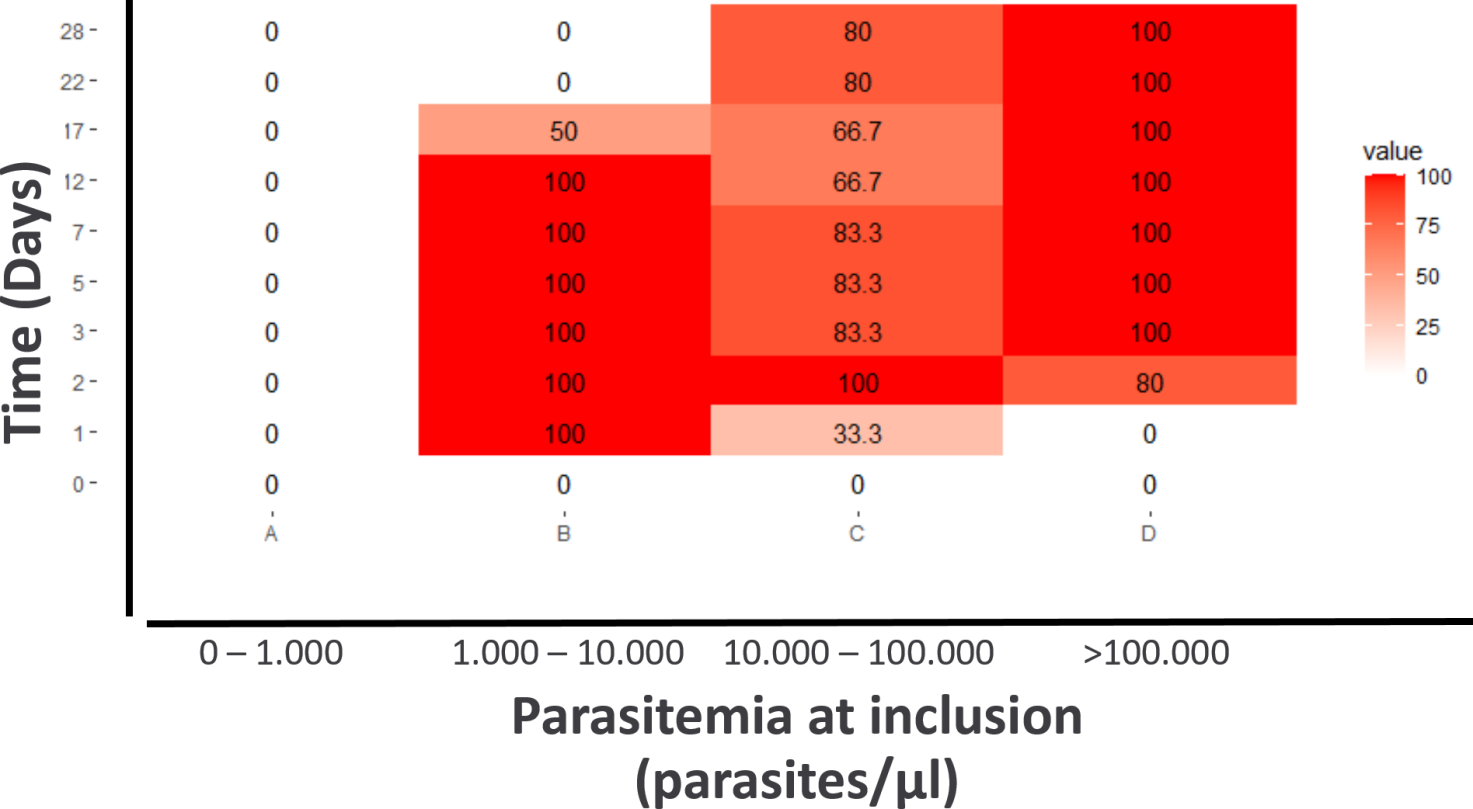
FP rate of the HRP2-RDT Paracheck-Pf for each time point stratified by groupings of participants who showed different ranges of initial parasitemia by TBS: (**I**) <1,000 parasites/µL (ii); 1,000 to 10,000 parasites/µL; (iii) 10,000 to 100,000 parasites/µL; and (iv) >100,000 parasites/µL. FP is expressed in percentage. A result of 100% FP represents a positive HRP2-RDT result while parasites are not detectable anymore. However, a 0% FP can have two interpretations, either there are detectable parasites for all participants within the group, resulting in a positive RDT test, or there are no detectable parasites in all participants in the group and the RDT test shows a negative result.

Obviously, the asexual stage parasites disappeared as determined by TBS at day 5 at the latest, whereas HRP2 levels remained positive for a long time period. The false-positive rate (FP) for HRP2-RDT results during follow-up was stratified according to groups with varying levels of thick blood smear (TBS) parasitemia prior to treatment initiation. (Fig. 4; Fig. S6). Gametocyte carriers after treatment and reappearance/reinfections were excluded from this estimation. As defined in the study inclusion criteria, participants at inclusion showed 0% FP. For participants exhibiting low parasitemia upon inclusion (<1,000 parasites per µL), the FP rate remained at 0% throughout the study period. This observation was made as these individuals attained parasite clearance, leading to consistently negative results on RDTs. Conversely, participants with higher levels of parasitemia upon inclusion (>100,000 parasites per µL) exhibited a prolonged period of 100% FP rate after parasite clearance from circulation. Consistent with the results of HRP2 ELISA, persistent long-term HRP2 positivity in RDTs was highly correlated with initial parasitemia (Paracheck-Pf RDT Spearman *P* < 0.001, correlation coefficient 0.6; BinaxNow T2 band Spearman *P* = 0.001, correlation coefficient 0.6).

## DISCUSSION

The work outlined here shows for the first time quantitative circulating HRP2 levels for 28 days after treatment of Pf mono-infections in humans from a hyper-endemic area. In our work, we propose a two-compartmental elimination model to describe the kinetics of the protein in the peripheral blood, which shows an elimination slope coincident with parasite elimination during the first 3 days and a subsequent slower terminal elimination slope until day 28.

The data suggest that the HRP2 persists in circulation long after parasite clearance, confirming previously published data (22, 26, 31, 40, 41). In acute malaria infections, the parasitemia at treatment initiation reflects the prolonged HRP2 clearance (42–46). According to reported cases, HRP2 can remain detectable for at least 7 days (45, 47) and even 32 days (44) after a successful treatment. In this study, seven participants who had no recognizable gametocytes in their blood after treatment and also had neither reinfection nor recrudescence were still positive on day 28 for HRP2. Their HRP2 values correlated well with the initial parasitemia.

The plasma half-life previously described by others is 3.67 days and the half-life in whole blood is 3–5 days, which takes only a constant decay of HRP2 into account (24, 28, 29, 48). These estimates do not adequately address the reported false positivity rate of HRP2-based RDTs long after treatment. However, the terminal elimination half-life using a two-compartment model seems better suited to explain the long positivity of RDTs after cured parasitemia. Thus, the half-lives previously calculated by others might have been underestimated (22, 24, 26, 28, 29, 41, 49).

The half-life of the terminal phase is the most frequently reported parameter in pharmacokinetics when determining the appropriate dose in repetitive administrations, considering the accumulation of the drug (50). The clearance and distribution of HRP2 in the body are maintained in equilibrium between the HRP2 in plasma and HRP2 in a second body compartment. The second compartment could be explained based on recent observations that a high number of HRP2-containing red blood cells remain in the bloodstream after treatment (40). The antigen expression of these initially infected erythrocytes is perpetuated after the process of erythrocyte pitting in the spleen, in which parasites are removed from the RBC, and these once-infected RBCs return to circulation still containing HRP2 in the cytoplasm. Thus, this can be attributed, in part, to the slower clearance of HRP2 during the second phase of elimination from the erythrocyte fraction of whole blood. This might explain a high false positivity rate from blood samples once the parasites are cleared.

Interestingly, the concentration of HRP2 in the peripheral blood increased slightly after treatment. Parasitemia estimation using microscopy or qPCR only considers the non-sequestered parasites. The increase in HRP2 on the following day after treatment initiation in seven participants might be coming from previously sequestered, dying parasites, which release HRP2 into the plasma, but remain partially engulfed in pitted cells (51, 52). Assuming some of the parasites were sequestered, when treatment was initiated they were eliminated by direct parasite killing with the effect of inhibition of parasitic replication and transcription activity, as well as pitting, thus creating a sudden increase in hollow previously infected erythrocytes still containing HRP2. Interestingly, it has recently been reported that pitting is more likely to occur when artemisinin-based combination therapies are used, as we did in our study (53). Future models should incorporate the spleen’s clearance effect under different therapies to provide a more accurate representation of HRP2 kinetics, for example, by estimating the cell number of pitted erythrocytes by flow cytometry and differentially quantifying the protein both in the serum and in the erythrocytes separately.

In cases of severe malarial infection, circulating HRP2 levels have previously been stressed to represent the sequestered *P. falciparum* mass (54). Data collected from a previous study in Lambaréné (55) reflected that higher amounts of HRP2 were detected in children suffering from severe malarial anemia (data not shown), a complication of the disease associated in the literature with a “chronic” infection state (56). This strongly supports the hypothesis that HRP2 accumulates over time in these patients with prolonged parasitemia or repetitive infections. Thus, HRP2 levels can be regarded as a marker for cumulative parasitemia over time.

Artesunate/amodiaquine remains one of the first line of treatment in children with acute malaria in Gabon (57–59). Artesunate acts in the early stages (60) and amodiaquine is a potent schizonticide (61) and the combination has an effect on gametocytes (62, 63). As in our study, a 3-day course treatment with this combination leads to microscopically undetectable asexual parasites and treatment failure is lower than other artemisinin combination therapies (ACTs). Lambaréné is a meso- to hyper-endemic region where malaria is perennial and reinfections are usual. Due to the small study sample size, the only single treatment failure (after genotyping (38) correction) conferred a slightly higher proportion of treatment failure compared to previous reports (64–67) under the same posology. Data from Gabon gathered by others show amodiaquine resistance of 28.2% (68–70).

HRP2 has been linked to hemoglobin metabolism as a method for the parasite to digest hemoglobin as a resource for protein synthesis (18). However, late-stage gametocytes do not consume hemoglobin, and it has been suggested that especially late stages might lack HRP2 expression (25, 71). Hanssen et al. (72) showed that hemoglobin digestion stops at gametocyte stage IV as hemoglobin crystals do not change in size (72). Despite other possible factors, a correlation of HRP2 antigenemia during convalescence has previously been shown in patients presenting gametocyte-positive films (26). Circulating gametocytes influencing HRP2-RDTs results has not been well investigated to date.

Sub-detectable parasitemia has been suggested to contribute to positive RDTs (73). A model estimated that about 8 parasites/μL were necessary to maintain a positive RDT in chronic infection (45). Artemisinin dormancy phenomenon may play a role in recrudescence cases which may contribute to maintaining certain levels of HRP2 detectable by especially ultrasensitive HRP2-based RDTs (20, 74). However, evidence for the possible influence of long-term parasitemia below the usual detection threshold on HRP2-RDT positivity is scarce. Based on HRP2 levels prior to treatment and those seen at reinfection/reappearance, HRP2 accumulation reaches significant levels over several cycles of asexual growth. Thus, sequestration alone might not be the cause of HRP2 elevation. As can be intuitively seen from severe malarial anemia, repetitive or prolonged infection could also be necessary to reach elevated levels of HRP2 accumulation in the body. Most research on HRP2 kinetics has been conducted on mild malaria cases, and severe cases have not been extensively investigated until today.

One limitation of the study was to use only whole blood samples and not additional plasma. However, ELISA HRP2 and RDT results are congruent with each other. Whole blood is the sample of choice for RDTs. In our study, RDTs detected HRP2 slightly better than ELISA, as some RDTs were still positive when ELISA results were below the detection limit. The sample size for the half-life calculation was also limited, as some participants had gametocytes, recrudescence/reappearances, and the detection limit of the ELISA hampered the HRP2 measurements in some participants.

### Conclusions

The HRP2 burden in the non-severe malaria cases in the present study represents well the circulating parasites before treatment. However, as reflected by the terminal half-life calculated here, HRP2 may persist for a long time in the body and, under frequent, recurrent, prolonged, and/or high parasitemia without efficient treatment, may accumulate. This might contribute to reducing the specificity of the HRP2-RDTs, which remains a concern, especially in endemic areas where children are vulnerable to malaria and other infections.

Before refining the design and calibration of RDTs to increase their sensitivity, a more precise description of the kinetics of HRP2 elimination is indispensable. This can provide valuable insights into the diagnosis and management of malaria, especially in terms of therapeutic strategies.

Thus, each case should be evaluated based on the patient’s history before proceeding with further measures. Understanding the kinetics of HRP2 clearance holds significant implications for interpreting RDT outcomes and malaria surveillance efforts.

## Data Availability

The data supporting this study may be made available to the public upon reasonable request through the corresponding author (rolf.fendel@uni-tuebingen.de).

## AFTER EPUB

This article was published on 28 August 2024 with an error in the affiliations. The affiliations were corrected in the current version, posted on 10 September 2024.

## SUPPLEMENTAL MATERIAL

Supplemental figures and tables spectrum.00994-24-s0001.pdf Fig. S1 to S6; Tables S1 and S2.

ASM does not own the copyrights to Supplemental Material that may be linked to, or accessed through, an article. The authors have granted ASM a non-exclusive, world-wide license to publish the Supplemental Material files. Please contact the corresponding author directly for reuse.
