# Supplementary material for: Slow clearance of histidine-rich protein-2 in Gabonese with uncomplicated malaria
Source: Microbiol Spectr. 2024 Aug 28;12(10):e00994-24. doi: 10.1128/spectrum.00994-24 (PMC11449231; doi:10.1128/spectrum.00994-24)
Supplement: Supplemental figures and tables — Fig. S1 to S6; Tables S1 and S2. [file spectrum.00994-24-s0001.pdf]

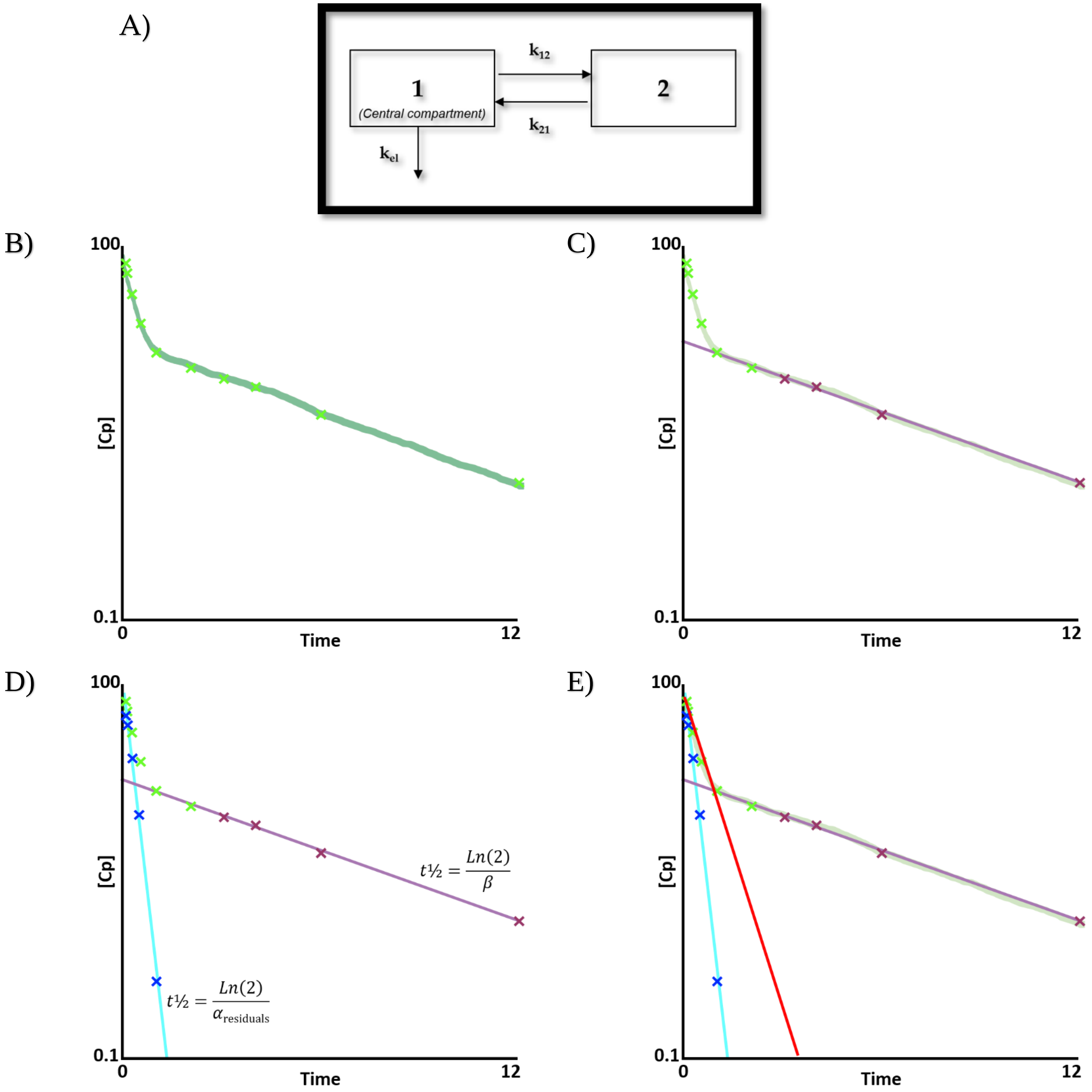

Supplemental figure 1. Representation of a two-compartment model. A) In the elimination model, there is a constant for the elimination (kel) and two constants for the continuous movement of compound between the central compartment (circulation) and second compartment (other parts of the body). The rate constants k12 and k21 represent the first-order rate transfer constants for the movement inward (k12) and outward (k21) of the compound to compartment "2". Most two-compartment models assume that elimination occurs from the central compartment "1". B) After and equilibrium is achieved, the linear regression shows a linearization in the semilogarithmic plot of the concentration (y-axis) and the days (x-axis). C and D) The slope of the regression line of these points (purple line) produces a slope used to calculate the half-life with the formula t1/2=Ln(2)/β. By resting the real values during the initial time points to the extrapolated ones (called residuals) from the purple line, new values are generated (blue points) that help to generate a new regression line (blue line). With the generated slope, the half-life of the residuals can be calculated. E) This half-life is closer to the actual elimination

during the onset of the elimination curve than the half-life that could be generated from a linear regression (red line) of the original values (green crosses), since it takes into account the contribution of the terminal elimination.

A)

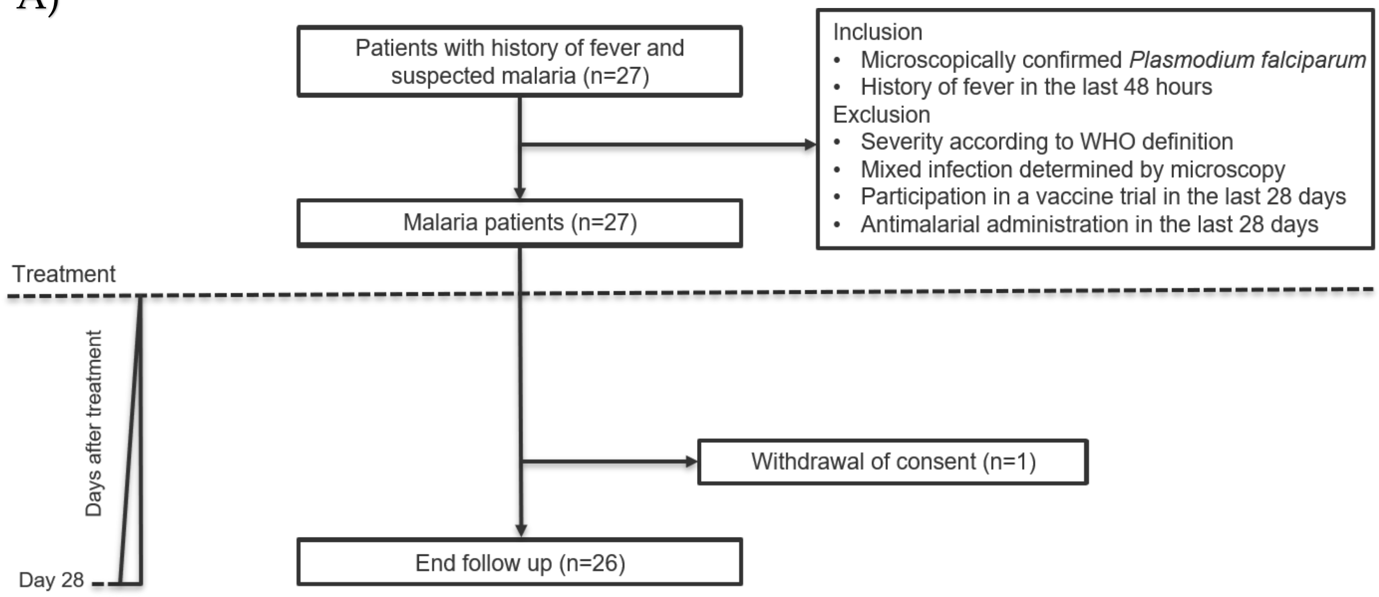

B)

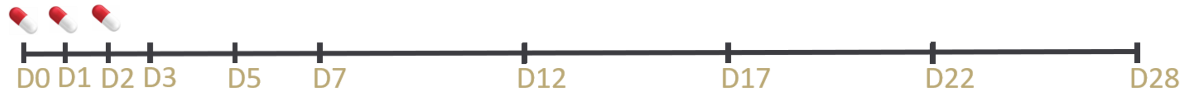

Supplemental figure 2. Study design, treatment and follow-up schedule. A) Participants enrolled arrived at the Albert Schweizer Hospital with mild malaria symptoms and, were treated with a 3-day course of artesunate/amodiaquine starting at day 0. B) Participants were followed-up for 28 days on days 1, 2, 3, 5, 7, 12, 17, 22, and 28.

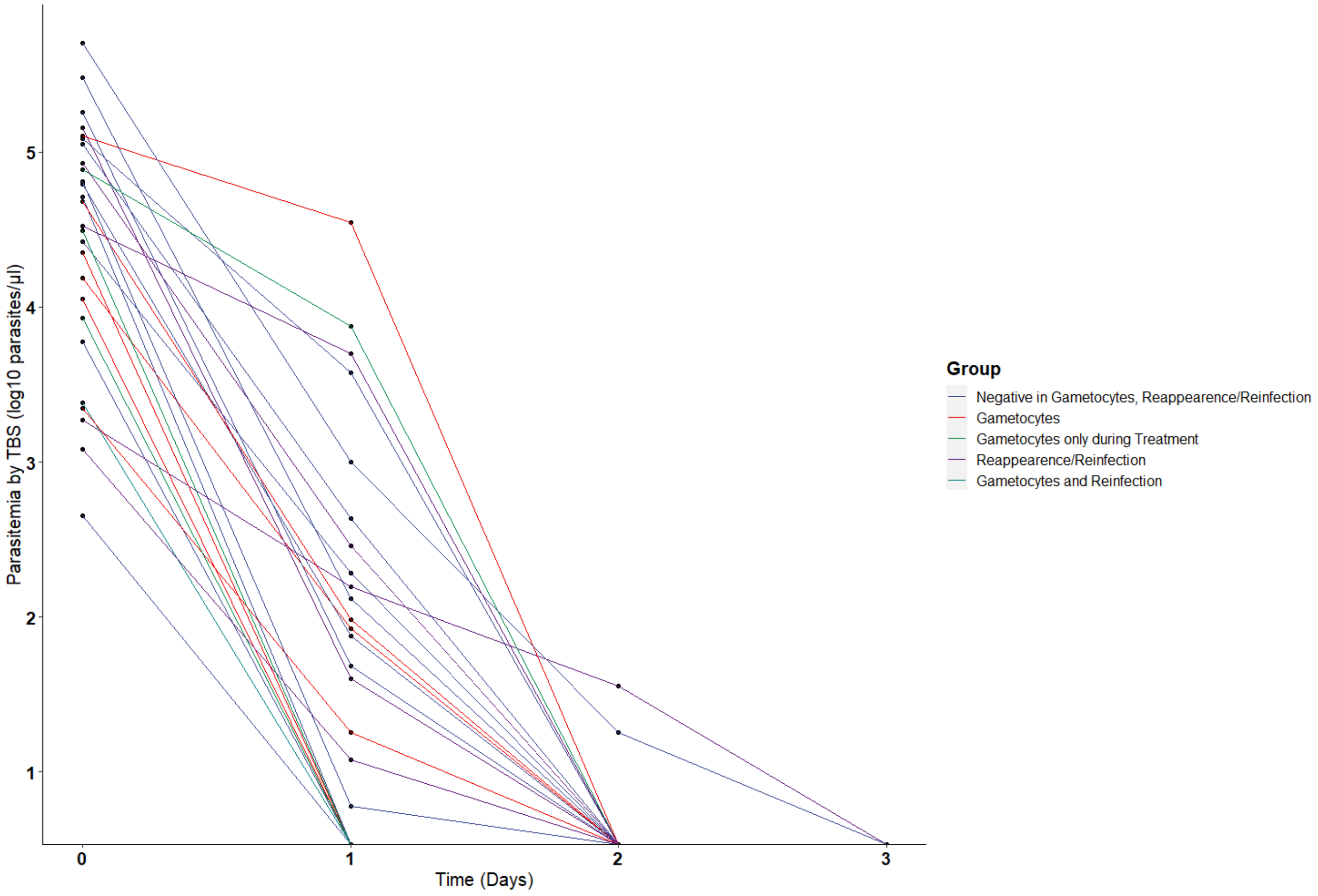

Supplemental figure 3. Parasite clearance by microscopy from first artesunate/amodiaquine administration. The kinetic of only the asexual parasitemia are shown as estimated by TBS for the first three days of treatment. The number of parasites detected was calculated as log10 (asexual parasites per µL). Circulating gametocytes detected by TBS are not shown here.

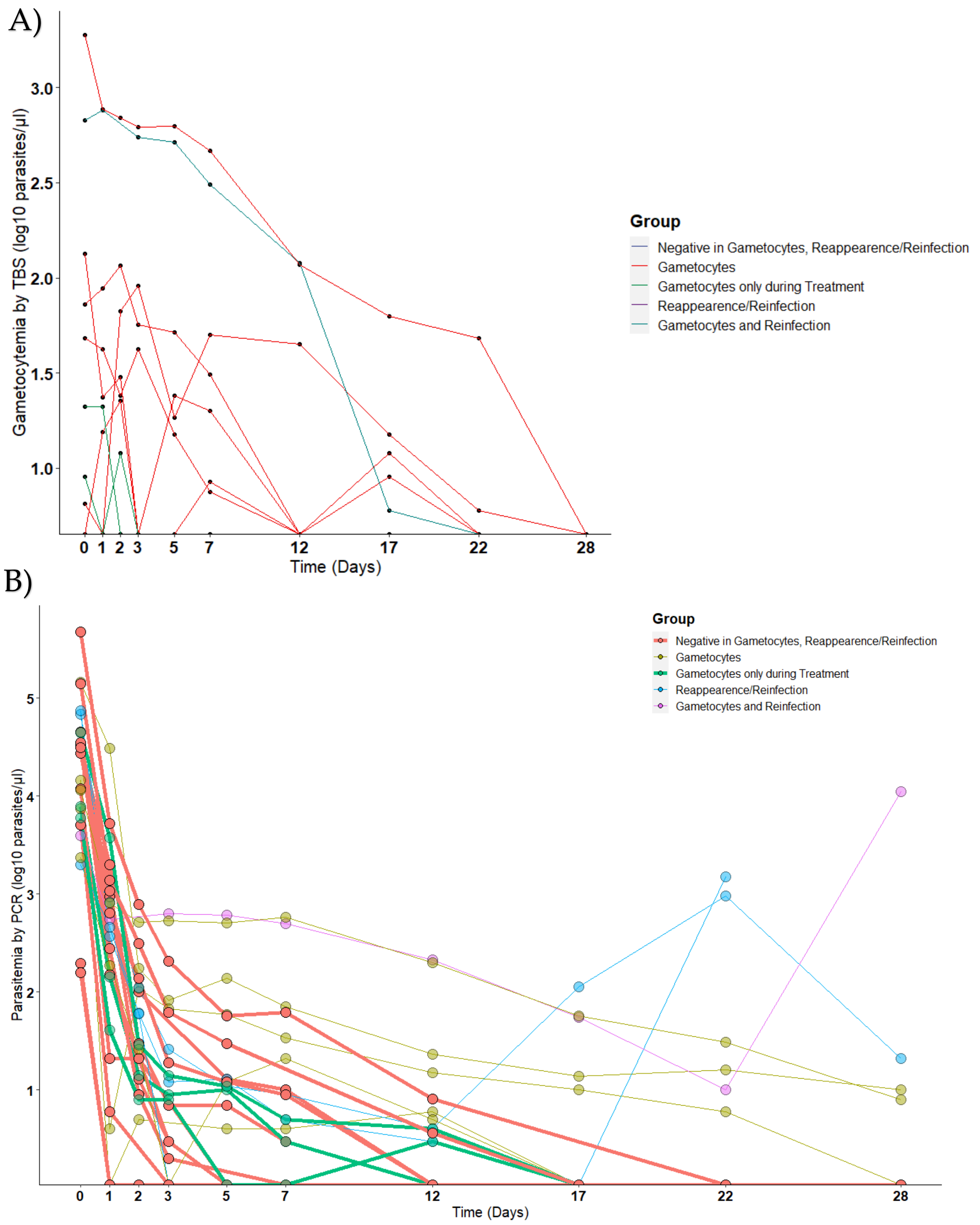

Supplemental figure 4. *P. falciparum* gametocytes were investigated during the trial at the sampling days 0, 1, 2, 3, 5, 7, 12, 17, 22 and 28. A) Gametocytes were detected microscopically on blood slides (TBS) during the course of the trial. The number of parasites detected was calculated as log10 (gametocytes per µL). B) Gametocytes by qPCR cannot be distinguished with the technique used from asexual stages. Parasites remained detectable for gametocyte carriers and also reinfections and reappearances showed an identifiable increase in parasitemia by qPCR. For the selected group for the HRP2 analysis, the last detected parasites were on day 12.

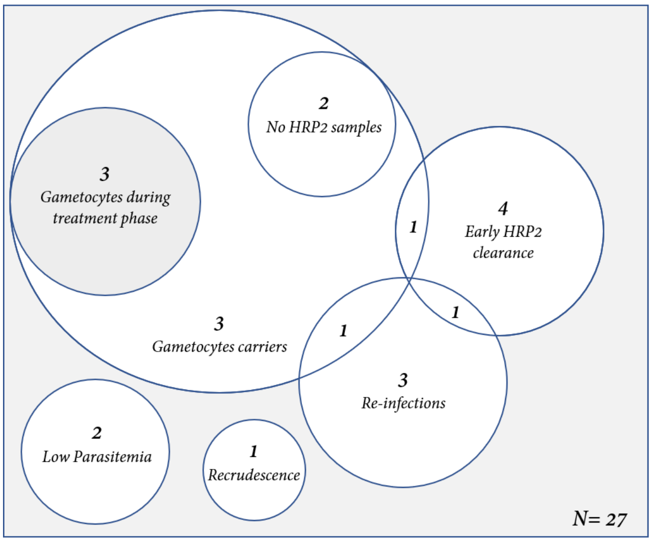

N = 27

- 2 Low Parasitemia
- 2 No HRP2 Samples + Gametocytes carriers
- 3 Gametocyte carries
- 1 Gametocyte carries + Re-infection
- 1 Gametocyte carries + Clearance of HRP2 before day 12
- 1 Re-infection + Clearance of HRP2 before day 12
- 3 Re-infections
- 4 Clearance of HRP2 before day 12
- 1 Recrudescence

18 Sum (eliminated from the analysis)

9 Remained

Supplemental figure 5. Venn diagram showing different groups of participants. Eligible participants for calculation of the terminal half-life (in gray). Of an initial 27 participants, 18 were excluded for different reasons, resulting in only 9 remaining.

A) PCR

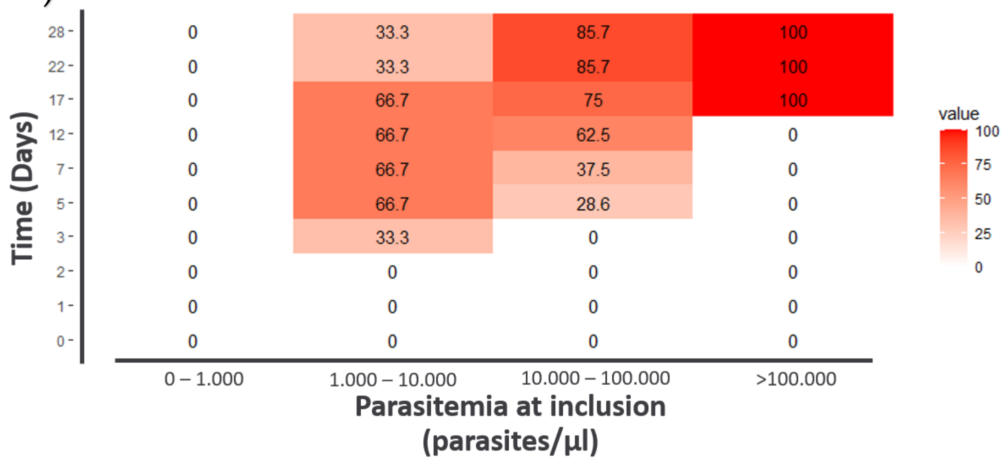

B) Microscopy

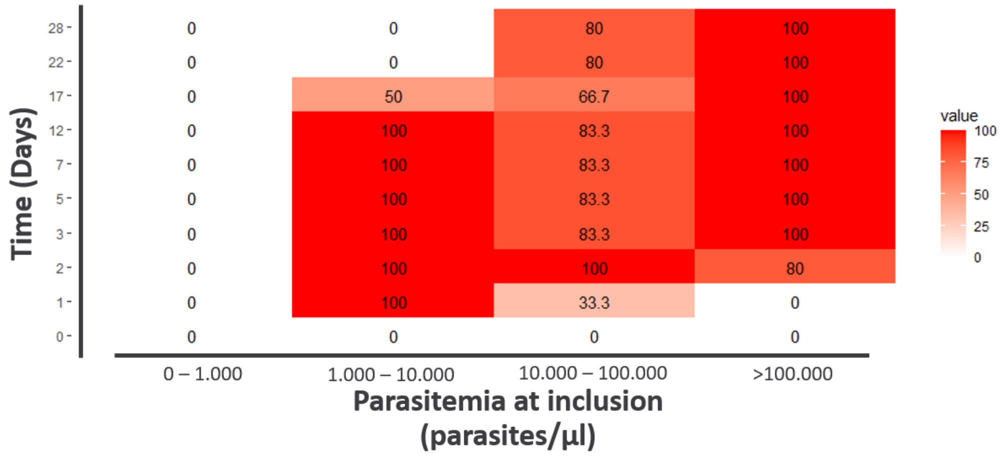

C) PCR

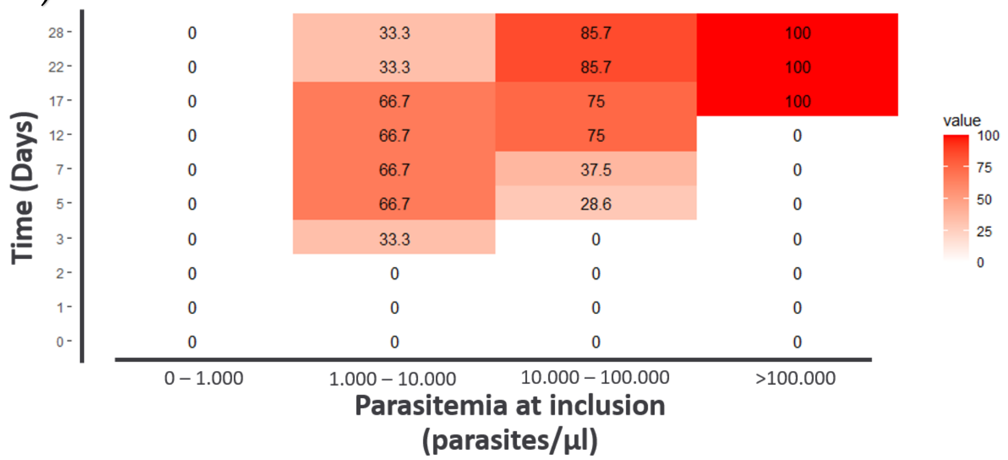

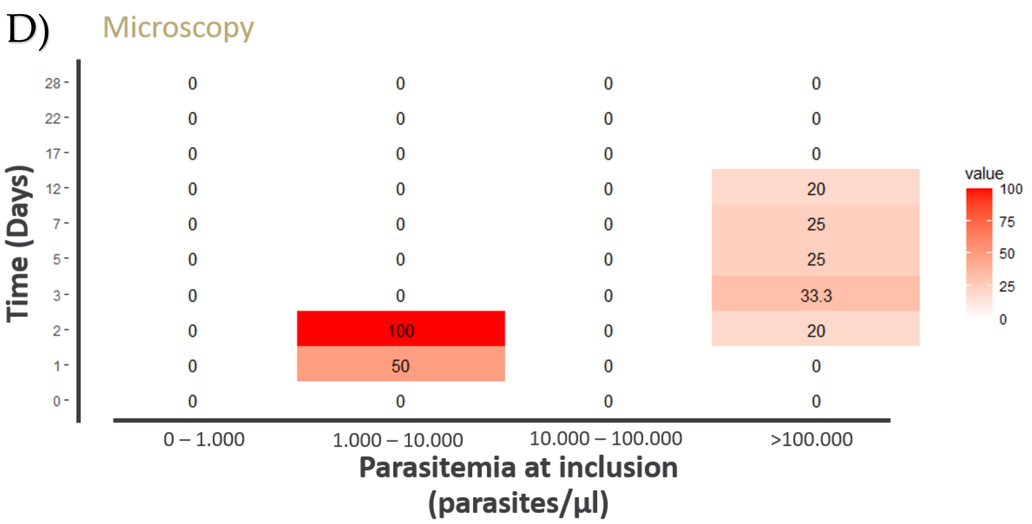

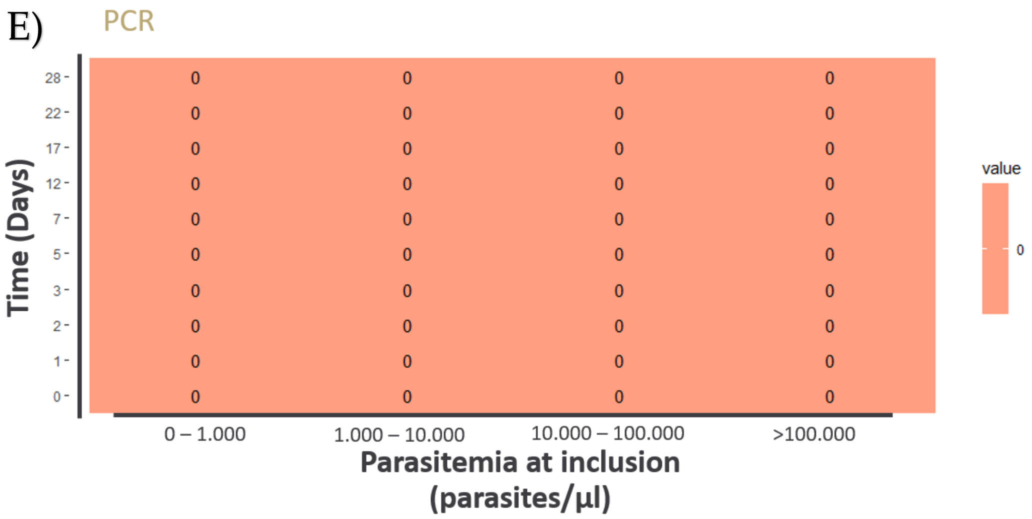

Supplementary figure 6. Performance of Paracheck-Pf and BinaxNow RDTs in terms of their false positivity rate (FP) to different parasitemias measured by qPCR or microscopy. Paracheck-Pf detects only HRP2 while BinaxNow RDTs detect pan-*Plasmodium* aldolase antigen (T2 band) along with HRP2 (T1 band). Additional to the FP shown in figure 4 we represent here: A) FP for Paracheck^{TM}Pf® stratified by different parasitemias measured by qPCR; B) FP for BinaxNow T1 band when compared to microscopy C) or by qPCR; and D) FP for BinaxNow T2 band calculated from parasitemias by microscopy E) or by qPCR.

Supplementary table 1. PCR reactions for simultaneous detection. Combined analysis was performed for *P. falciparum*/Ph-HV-1 or *P. falciparum*/*P. malariae*/PhHV-1 or *P. ovale*/*P. vivax*/PhHV-1, respectively. Fluorophores and quenchers were used as indicated and can deviate from the given reference. For *Plasmodium spp.* one single primer pair was used (Producers: 1 Operon Biotechnologies, Germany; 2 Applied Biosystems, USA).

Target Organism	Primer / probe name	Primer / probe sequence	Producer	Reference
P. falciparum	Plas-7F	GTTAAGGGAGTGAAGACGATCAGA	1	https://doi.org/10.1006/mpev.1996.0068
	Plas-171R	AACCCAAAGACTTTGATTTCTCATAA	1	https://doi.org/10.1371/journal.pone.0052719
	PfalMGB	[FAM]CTTTCGAGGTGACTTTTAGAT[NFQ]	2	https://doi.org/10.1007/s00436-007-0804-4
P. malariae	Plas-7F	(as above)		https://doi.org/10.1007/s00436-007-0804-4
	Plas-171R	(as above)		
	MGBmalariae89	[VIC]AGCTATCTAAAAGAAACACTCAT[NFQ]	2	
P. ovale	Plas-7F	(as above)		https://doi.org/10.1007/s00436-007-0804-4
	Plas-171R	(as above)		
	MGBovale90	[FAM]CCCGAAAGGAATTTTCTTATT[NFQ]	2	
P. vivax	Plas-7F	(as above)		https://doi.org/10.1007/s00436-007-0804-4
	Plas-171R	(as above)		
	MGBvivax133	[VIC]TTTTCTCTTCGGAGTTTATT[NFQ]	2	
PhHV-1	PhHV-267s	GGGCGAATCACAGATTGAATC	1	https://doi.org/10.1016/S1386-6532(02)00197-X
	PhHV-337as	GCGGTTCCAAACGTACCAA	1	
	PhHV-305tq	[Cy5]TTTTTATGTGTCCGCCACCATCTGGATC [BHQ3a]	1	

Supplemental table 2. Study Demographics.

		**Study participants (n = 27)**
Sex	male n (%)	8 (29)
	female n (%)	19 (70)
Age	Mean (SD)	8 (5)
	Median (range)	7 (1 - 23)
Weight (Kg)	Mean (SD)	28 (18)
	Median (range)	21 (8 - 68)
Height (m)	Mean (SD)	1 (0.3)
	Median (range)	1 (1 - 2)
Pyrexia (Days)	Median (range)	2 (1 - 14)
Splenomegaly	n (%)	4 (15)
